# Case report of the successful treatment of pemphigus vulgaris using ovine forestomach matrix graft

**DOI:** 10.1093/jscr/rjae848

**Published:** 2025-01-14

**Authors:** William A Kokal, Jessica Simon

**Affiliations:** Department of General Surgery, Lee Wound Care & Hyperbaric Medicine, Fort Myers, FL 33912, United States; Department of Medical Affairs, Aroa Biosurgery Limited, 64 Richard Pearse Drive, Auckland 2022, New Zealand

**Keywords:** pemphigus vulgaris, extracellular matrix, ovine forestomach matrix, Stevens-Johnson syndrome, toxic epidermal necrolysis

## Abstract

Pemphigus vulgaris (PV) is a subtype of pemphigus and life-altering disorder that results in the formation of intraepithelial blisters in mucosa and skin. Though the etiology is not well understood, it is an autoimmune disorder resulting in acantholytic blisters due to auto-antibodies targeting proteins of keratinocyte adhesion. Rapid diagnosis and restoration of the epidermal layer is imperative for patients with PV as widespread epidermal damage can lead to high morbidity and mortality rates. This case report presents the treatment of PV in a 53-year-old female who presented after 9 months of worsening symptoms and 30% total body surface area blistering. Most of the lesion was re-epithelialized in 1 week, with complete healing in 4 weeks following a single application of ovine forestomach matrix (OFM) graft. This case represents the first report of the use of OFM to aid regeneration of epithelial lesions resulting from an autoimmune bullous disease.

## Introduction

The autoimmune bullous diseases (AIBDs) represent a group of pathologies characterized by loss of function of basement membrane and adhesion proteins resulting in blistering or bullae of the epidermal layer. AIBDs include pemphigus vulgaris (PV), pemphigus foliaceous, bullous pemphigoid, epidermolysis bullosa acquisita [1] and present similarly to Stevens-Johnson syndrome (SJS) and toxic epidermal necrolysis (TEN) [2], complicating the diagnosis. Rapid diagnosis of the disease and therapeutic intervention help prevent disease progression, and when left untreated can result in malnutrition, dehydration, sepsis, and death [3]. Corticosteroids and/or Rituximab remain first-line treatments for PV and have shown to be effective in halting disease progression [4], while high-potency topical corticosteroids are often deployed to manage persistent lesions [5]. Patient quality of life is most often directly correlated to absence of blistering and improvement of symptoms [3].

While therapeutic interventions may halt disease progression, regeneration of the missing or damaged epidermal layer in PV patients remains a challenge, with reconstruction being managed within the burn unit. The following case report describes the reconstruction of a PV patient with a 30% total body surface area (TBSA) epithelial injury using an ovine forestomach matrix (OFM) graft which was selected due to its known ability to inhibit wound proteases [6].

## Case report

**Figure 1:**
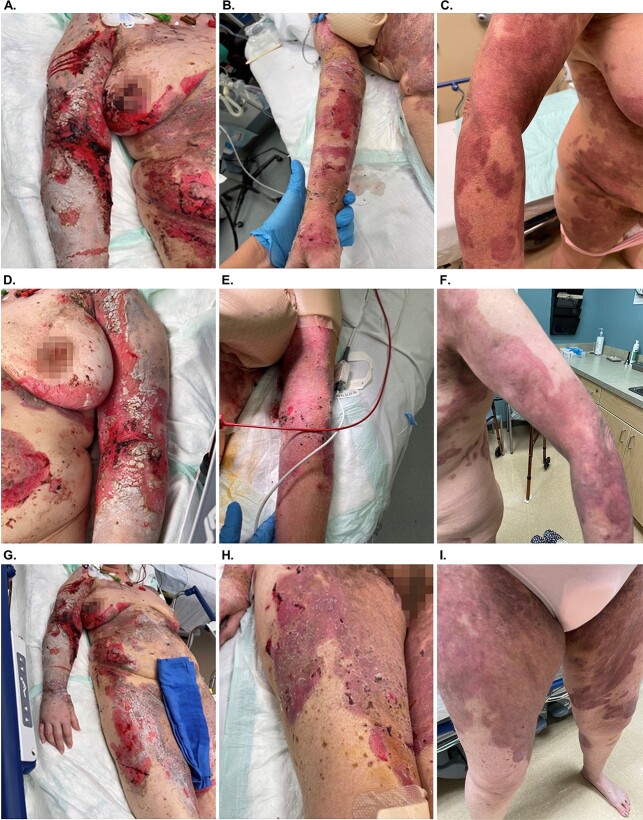
Treatment of PV with OFM graft to the upper right extremity (A–C), upper left extremity (D–F) and right thigh (G–I). Image sets show progression at treatment initiation (A, D, and G), at 1 week post application of OFM (B, E, and H) and at 4 weeks post application of OFM (C, F, I).

A 53-year-old female with no prior autoimmune disease or significant medical history presented following 9 months of worsening symptoms and painful lesions. Initial treatments with oral antibiotics failed and lesions continued to develop and deteriorate. Clinical exam demonstrated ⁓3000 cm^2^ of blistering/open wounds across multiple body areas resulting in 30% TBSA of epithelial injury (Fig. 1A). Initial blood work demonstrated ESR 40 (H) and CRP 7.8 (H). Specimen histology confirmed PV. Due to the extent of the affected areas and patient pain, reconstruction was staged to different areas of the body, treating one area at a time ⁓1 week apart. In the operating room, the lesions were gently debrided and a 3-layer OFM graft (Myriad Matrix^TM^, Aroa Biosurgery Limited, Auckland, New Zealand) was applied to the epithelial injuries. The graft was rehydrated with sterile saline, then dressed with a contact layer (Versatel®, Medline Industries), followed by gauze and tape. Areas adjacent to the OFM graft that were not ready for treatment yet were dressed with topical mupirocin and gentamycin, followed by gauze and tape. At post-operative week one, residual OFM graft was gently removed from the treatment areas to reveal epithelialized tissue. Adjacent untreated areas demonstrated minimal healing progress. The remaining two treatment areas received a single application of OFM graft on subsequent weeks. Right upper extremity tissue was fully epithelialized at 1 week after OFM application (Fig. 1B), while the left upper extremity and lower extremities demonstrated small areas of open wounds and were otherwise epithelialized (Fig. 1E and H). By treatment week four (Fig. 1C, F and I), full epithelialization was achieved across all treatment areas. Patient was discharged from the hospital 2 days later.

## Discussion

Pharmacologic treatments for AIBDS and SJS/TEN are well documented [4]; however, limited literature exists on reconstructing epithelial injuries post-stabilization. Since the lesions represent only epidermal loss, products to accelerate re-epithelialization have the potential to reduce infection risk, a primary cause of mortality among this patient group, often exacerbated by corticosteroid therapy [7]. While utilization of collagen- and extracellular matrix (ECM)-based devices is widespread for the treatment of wounds [8], reports describing the use of these advanced wound care products to accelerate epithelial regeneration in AIBDs and SJS/TEN are scarce in the literature. Currently, reports on advanced wound care products for the management of AIBDs and SJS/TEN predominantly focus on porcine xenografts [9] and a biosynthetic product, Biobrane®, comprising reconstituted porcine collagen and a flexible silicone sheet. Studies in SJS/TEN have reported a mean healing time of 13 days [10] and a significant reduction in fluid loss, pain scores, and corresponding pain medication [9]. Bhattacharya *et al.* [11] reported the use of a reconstituted collagen dressing on seven TEN patients to aid re-epithelialization of the lesion.

To the authors knowledge the current report is the first published description of an advanced ECM-based device being utilized to aid epithelial regeneration in AIBDs, and more specifically PV. Unlike reconstituted collagen products (e.g. Biobrane®), OFM is a decellularized ECM and has been widely adopted for the treatment of complex wounds and for soft tissue reconstruction [12]. Most typically, OFM is used to scaffold the regeneration of well vascularized tissue, whereby the device undergoes cell infiltration, re-population and neovascularization. In the current report, the OFM graft was used to cover and protect the PV lesion and aid re-epithelization. Studies have previously shown OFM to be effective in the re-epithelialization of superficial and partial-thickness burns [13]; however, this is the first report of use in the treatment of AIBDs. Epithelization occurred in ⁓1 week, which is consistent with previous studies using the biosynthetic product Biobrane® in the treatment of SJS/TENS (<2 weeks) [10]. Pain is especially debilitating for AIBDs and SJS/TEN patients [9]. The patient reported a marked reduction in pain once OFM was introduced to the treatment regime. This observation is consistent with previous studies that reported pain reduction following use of porcine xenograft in the management of AIBDs and SJS/TEN lesions [9].

OFM is comprised of many of the matrisomal proteins that exist in tissue ECM, including but not limited to structural, signaling, and adhesion proteins [14]. For example, OFM contains the anti-inflammatory protein, tissue inhibitor of metalloproteinase (TIMP)-4 [14] and has been shown to inhibit various tissue proteases (e.g. matrix metalloproteinases (MMPs) and neutrophil elastase) *in vitro* [6]. The involvement of wound proteases in PV lesions likely contributes to the slow rate of re-epithelisation by limiting cellular attachment required for keratinocyte migration and proliferation [15]. The ability of OFM to target and inhibit MMP activity may provide a more mechanistic based approach to re-epithelializing AIBDs and SJS/TEN lesions.

The current case describes the first reported use of OFM for epithelization of PV lesions representing a 30% TBSA. Most of the de-epithelized areas had closed within 1 week and the patient reported a dramatic improvement in pain. Additional studies are warranted to explore application of OFM to the treatment of life-threatening AIBDs and SJS/TEN lesions.

## Data Availability

No new data was generated or analyzed in support of this research.
